# Optimization of Two Eco-Friendly Extractions of Black Medick (*Medicago lupulina* L.) Phenols and Their Antioxidant, Cosmeceutical, α-Glucosidase and α-Amylase Inhibitory Properties

**DOI:** 10.3390/molecules26061610

**Published:** 2021-03-14

**Authors:** Lejsa Jakupović, Marko Kalvarešin, Karla Bukovina, Valentina Poljak, Lovorka Vujić, Marijana Zovko Končić

**Affiliations:** 1 Department of Pharmacognosy, University of Zagreb Faculty of Pharmacy and Biochemistry, Marulićev trg 20, 10 000 Zagreb, Croatia; ljakupovic@pharma.hr (L.J.); m.kalvaresin@gmail.com (M.K.); karla.bukovina@gmail.com (K.B.); vpoljak23@gmail.com (V.P.); 2 Department of Food Chemistry, University of Zagreb Faculty of Pharmacy and Biochemistry, Domagojeva 2, 10 000 Zagreb, Croatia; lvujic@pharma.hr

**Keywords:** eco-friendly extraction, glycerol, *Medicago lupulina*, polyphenols, response surface methodology

## Abstract

*Medicago lupulina* is an ancient edible plant from the Fabaceae family. In this work, two eco-friendly methods for extraction of bioactive phenolics from *M. lupulina* were developed using mixtures of water with two non-toxic, skin- and environmentally-friendly polyol solvents: glycerol and polypropylene glycol. Ultrasound-assisted extractions were optimized using a Box–Behnken design. The independent variables were the concentration of organic solvent in water (X_1_), extraction temperature (X_2_) and time (X_3_), while the response was phenolic content. The optimum conditions for extraction of polyphenols were (X_1_, X_2_, X_3_): (45%, 70 °C, 60 min) and (10%, 80 °C, 60 min) for glycerol and polypropylene glycol extraction, respectively. The extracts prepared at optimum conditions were rich in phenolic compounds, mainly derivatives of apigenin, kaempferol, luteolin, quercetin, caffeic and ferulic acid, as well as coumestrol. Their cosmeceutical and antidiabetic activity was tested. Both extracts demonstrated notable antioxidant, anti-lipoxygenase and anti-α-amylase activity. In addition to those activities, the glycerol extract efficiently inhibited protein coagulation, elastase and α-glucosidase activity. Glycerol present in the extract displayed enzyme-inhibiting activity in several assays and supported the action of the bioactive constituents. Thus, the optimized glycerol extract is a desirable candidate for direct incorporation in antidiabetic food supplements and cosmeceutical products.

## 1. Introduction

The discovery, development and marketing of dietary supplements, the products containing dietary ingredients or their components and intended to supplement regular diet, are currently the fastest growing segments of the food industry [1]. Such supplements often include a wide range of plant-based products designed to display added nutrient value and presumed health benefits for numerous contemporary ailments. For example, type-2 diabetes mellitus is a growing public health concern related mostly to sedentary lifestyle and unhealthy eating habits. It is closely accompanied by states of oxidative stress and inflammation which further progress to secondary complications of diabetes such as cardiovascular disease, neuropathy, nephropathy and retinopathy. It has been found that those complications, as well as the states that induce them, may, at least partly, be managed by natural plant metabolites and supplements that contain them [2]. In addition to inclusion into dietary supplements, bioactive phytochemicals are increasingly being incorporated into cosmetics, and claims that a given product contains natural components are being especially appreciated by the consumers [3]. In such products they act as anti-inflammatory [4], anti-aging, as well as skin-whitening agents, to name a few [3]. 

Among numerous plant metabolites, phenolic compounds occupy one of the most prominent places. Their antioxidant and anti-inflammatory activity, as well as the ability to counteract various processes that occur under oxidative stress conditions, enables them to slow down aging, as well as to prevent or combat numerous contemporary degenerative diseases, including diabetes [5]. In addition, when taken by mouth, food phenolics, such as flavonoids [6] and phenolic acids [7], may come into contact and cause inhibition of gastrointestinal enzymes, including α-amylase and α-glucosidase, two enzymes responsible for break-down of starch and oligosaccharides, respectively. Reduction of complex carbohydrates digestion rates leads to better glycemic control and slows down progression of the disease. Furthermore, a growing body of research suggests that natural phenolics may counteract glucose-induced oxidative stress in diabetic patients, restore the levels of endogenous antioxidants in affected tissue and prevent or even reverse diabetic complications [2] such as neurological or ocular damage [6] 

Even though plant phenolics are most renowned for their role in health promotion, they are increasingly being used in cosmetic industry [5], where the antioxidant potential and their myriad biological activities render natural phenolics ideal preservatives and functional ingredients in natural cosmetics [8]. As a result, plants rich in flavonoids, phenolic acids, coumestans and other plant phenolics are increasingly being used in so-called cosmeceuticals, the products that share characteristics of pharmaceutical and cosmetic agents [9,10,11]. In such products they may be used as antioxidants and chelators of pro-oxidant metal ions protecting against peroxidation unsaturated fatty acids in creams and lotions [12]. Furthermore, their antioxidant activity may be displayed in skin where they protect macromolecules from exo-/endogenous free radicals and harmful solar radiation [13]. In addition, phenolic compounds may inhibit tyrosinase, the enzyme responsible for synthesis of skin-pigment melanin, whose uneven distribution may lead to various skin disorders such as solar lentigo and melasma [9]. Furthermore, they may also inhibit elastase, the enzyme whose increased activity is related to psoriasis, delayed wound healing, wrinkles formation and premature skin aging [14]. Finally, natural phenolics might act as anti-inflammatory agents. Their activity may be displayed through the inhibition of pro-inflammatory enzymes, such as anti-lipoxygenase (LOX) [15], as well as through inhibition of denaturation of tissue proteins [16]. 

In order to incorporate phenolics and other bioactive plants metabolites into nutritional, pharmaceutical and cosmetic formulations they must be effectively extracted from plant material. Ideally, the extraction should employ environmentally friendly, as well as cost-effective materials and solvents. One of the solvents that most readily fits into this description is glycerol (GL), a non-flammable, non-toxic liquid, that is a normal part of plant and animal metabolism. In addition, being a biodegradable solvent produced from renewable sources makes glycerol one of the most eco-friendly solvents for industrial applications [17]. Its use in various (semi)liquid pharmaceutical formulations is widespread due to its natural origin, high viscosity, low toxicity and humectant properties [18]. Thus, use of GL as solvent for UAE has an additional advantage that such extracts may easily be incorporated into the final product thus eliminating the time and energy consumption needed for solvent removal. Having in mind its widespread use, excellent biocompatibility and low impact on environment, the use of GL for extraction of natural compounds is relatively under-represented even among various eco-friendly solvents. Examples published so far, demonstrate that GL and GL/water mixtures were excellent solvent for phenolic antioxidants from licorice [19], *Echinacea purpurea* aerial parts [20], as well as onion [21]. However, while GL and GL/water mixtures are optimally suited for extraction of polar organic molecules, more lipophilic molecules are not so readily dissolved in such medium. 

Polypropylene glycol (PPG) is a biocompatible [22] and biodegradable [23] polymer that is a liquid at room temperature. Low molar weight PPG is freely soluble in water, but its solubility decreases with the increased molar mass. It is similar to polyethylene glycol (PEG), a polymer widely used for pharmaceutical and biomedical applications, but the replacement of a methyl group in the repeating ethylene glycol unit renders PPG more hydrophobic than PEG. PPG is a generally recognized as safe (GRAS) substance [24]. Due to its safety it has been approved for human injections and oral application, and as such is being widely used by the chemical, food, and pharmaceutical industries [22]. Its use in biphasic systems has shown that it is excellent solvent for extraction of natural and synthetic dyes [22,25]. Despite of its non-toxicity and humectant properties, as well as the fact that it is used in cosmetic products and as a food additive [24], the use of PPG/water mixtures for extraction of bioactive natural compounds has, to our best knowledge, not been recorded. 

*Medicago lupulina* L. (Fabaceae), is an annual biennial or short-lived perennial species native to Eurasia, often used in phytoremediation. The numerous, thin subsidiary roots of the plant form dense mats. Stems are up to 8 dm long, prostrate or erect, with trifoliate leaves [26,27]. *M. lupulina* is a traditional edible plant and rich source of various bioactive and nutritional compounds. The adult plants and its microgreens contain significant amounts of primary metabolites such as carbohydrates, including galactomannans [28] starch and soluble sugars, as well as proteins [29]. They are rich in triterpene saponins, mostly derivatives of soysapogenol and medicagenic acid [30]. Furthermore, aerial part of *M. lupulina* contain significant amount of various biologically active groups of phenolic compounds such as coumestanes, isoflavonoids and other flavonoids [29]. The flavonoids and other phenolic antioxidants in the plants were shown to be responsible for an excellent antioxidant, antiradical and reducing activity [26]. Furthermore, the published in vitro studies have shown that *M. lupulina* leaf extract has a pronounced antibacterial, antifungal and antitumor effect [31]. *M. lupulina* is closely related to *M. sativa*, well known medicinal plants used for the treatment of various disease related to oxidative stress such as arthritis, kidney disorders, as well as disorders of cardiovascular system [26]. However, while the activity of *M. sativa* is well known and recognized, the possible therapeutic applications of *M. lupulina* remain largely unknown.

The aim of this work was to optimize the ultrasound-assisted extraction (UAE) of bioactive phenols from the aerial parts of *M. lupulina* using mixtures of water with two non-toxic, water-soluble solvents: glycerol and polypropylene glycol. Additional goal was to test the biological activity of the prepared extracts using selected assays targeting the activities relevant to cosmeceutical and digestive tract-related anti-diabetic applications.

## 2. Results and Discussion

### 2.1. Extraction of Phenolics from M. Lupulina

In this work an eco-friendly UAE extraction of phenols from *M. lupulina* was optimized using a Box-Behnken design (BBD) and response surface methodology (RSM) (Table 1). 

The extraction conditions and the total phenol (TP) content of the extracts prepared in two BBDs are presented in Table 2. Both solvents were capable of dissolving phenolic compounds from *M. lupulina*, with notable similarities. The amount of extracted polyphenols differed greatly among extracts. In both designs, the extracts prepared by simultaneously using both the highest organic solvent content and the lowest extraction temperature (standard order 2) contained the lowest TP content. For example, Run 4 (standard order 2) in GL extraction contained only 98.86 μg/mL of phenolic compounds while Run 13 (standard order 14) of the same design contained as much as 248.12 μg/mL of phenols. PPG/water mixtures were somewhat less successful in dissolving phenolics, and their content ranged from 48.07 μg/mL to 167.71 μg/mL in Run 13 (standard order 2), and Run 14 (standard order 3) PPG-S3, respectively. In general, high temperature positively affected phenolic content of the extracts prepared by both solvents, especially at high concentrations of organic solvents. This is probably related to the reduction of the viscosity of organic solvents at higher temperatures, which is especially important in cases of highly viscous solvents such as GL [32]. 

**Figure 1:**
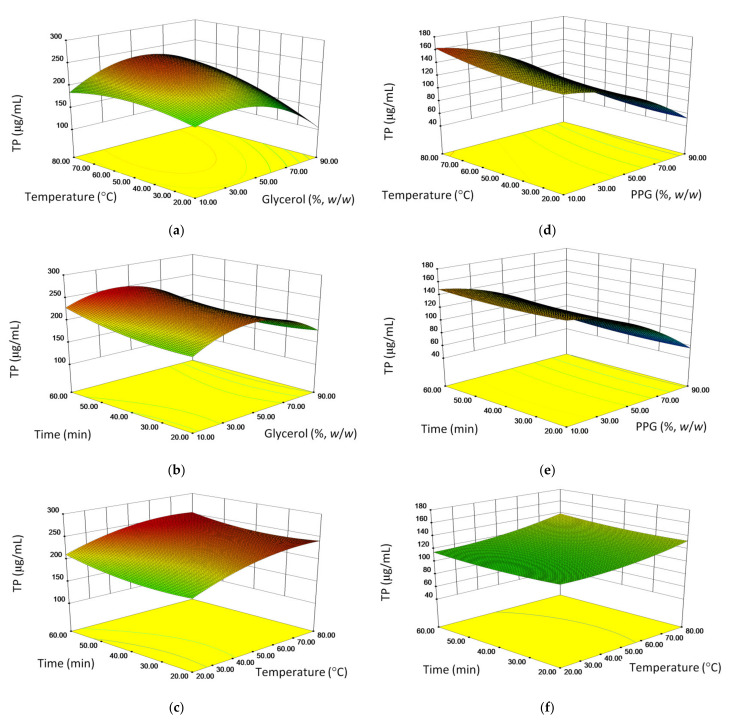
Influence of pairs of independent variables on phenolic content in two solvents: (**a–c**) glycerol; (**d–f**) polypropyleneglycol (PPG).

As presented in Table 3, multiple regression analysis of the experimental data, has shown that quadratic polynomial equations adequately explain the relationship between the response and the independent variables. In addition, for better visualization of the interactions of the experimental conditions and the obtained responses, the influence of the investigated UAE parameters on the TP in glycerol (Figure 1a–c) and PPG (Figure 1d–e) is presented in form of response surface plots.

The extraction conditions influenced the extraction in a relatively similar manner, although with a few differences. Overall, organic solvent content was the most important extraction variable. It negatively influenced phenolic content as a quadratic factor in both types of extraction. Thus, similar to other published examples [19], for both types of extraction there was an optimal organic solvent content at which the phenolic compounds were best extracted. In addition, organic solvent content influenced both types of extraction as a linear term. The linear influence was negative in both types of extraction. However, the absolute value of linear coefficient was much lower than the quadratic one in glycerol extraction, while for PPG extraction the opposite was true. Consequently, moderate GL content was better suited for the extraction of phenolic substances (Figure 1a,b), while for the same purpose low PPG content was more appropriate (Figure 1d,e). As the secondary phenolic metabolites are compounds of moderate polarity, this is related to relatively high hydrophobicity of PPG [22,25]. Thus, the use of PPG may reduce the need for the use organic solvents in mixtures intended for extraction of phenolic compounds. 

To a certain extent, a similarity between the two types of extraction was also reflected in the influence of the temperature on the yield of TP. For example, temperature as a positive linear term was a significant extraction variable in extraction by both solvents indicating that higher temperatures will be better suited for extraction. This is because high temperature in combination with ultrasonication may improve the extraction process by reducing the viscosity of the solvent and increasing kinetic energy of the molecules in the solutions [33]. Thus, a positive linear influence of temperature is generally expected and observed especially in case of extraction with GL and other viscous solvents [19]. In GL extraction, the temperature was also a significant quadratic negative term. However, the absolute value of quadratic coefficient was much smaller than that of the linear one. This indicates that for GL extraction there will be an optimal temperature, albeit relatively high, by which the extraction efforts are the most successful (Figure 1a,c). On the other hand, the higher temperature was generally more suited for PPG extraction (Figure 1d,f). There was also a positive interaction between glycerol content and temperature indicating that the high temperature positively contributes to extraction at high glycerol concentration. However, at low GL concentrations, low temperature is more appropriate. A slightly detrimental influence of the temperature on glycerol extraction may be attributed to its oxidation caused by hydroxyl radicals [34] whose production may be brought by ultrasonication at high temperatures [35]. Namely, in presence of hydroxyl radicals, glycerol is oxidized to glyceraldehyde and dihydroxyacetone [36]. They are known to react with natural phenolics, forming new condensed phenolic compounds and thus reducing the number of free phenolic groups available for reaction with Folin-Ciocalteu reagent [37]. Similarly, the interaction of glycerol concentration, temperature and higher ultrasonication power exerted a negative effect on the phenolic acids extraction from *Echinacea purpurea* aerial parts [20]. Such reaction was less likely in PPG due to the absence of primary hydroxyl groups and consequently lower reactivity [22]. In addition, combination of ultrasonication and high temperatures may also bring about generation of hydroxyl radicals in aqueous medium [35]. It has been found that they may subsequently react with phenolic substances, such as caffeic acid derivatives [38] thus reducing their concentration in the extracts prepared by ultrasonication. However, regarding a relatively high coefficient of the temperature as the linear term, as well as high yields of TP in GL extraction, the unfavorable influence of high temperature was rather weak. A slight inclination towards positive influence of longer extraction times may be observed in the graphs that depict time (Figure 1b,c,e,f). However, that influence did not reach statistical significance (Table 4). 

The analysis of variance (ANOVA), presented in Table 4, shows high F-values (>14) and low *p*-values (<0.01) of both models and indicates that they appropriately describe the relations between the independent and dependent variables. Furthermore, the insignificant values of lack-of-fit tests of both models (>0.05) confirms that the models are adequate to describe the experimental data. The relatively high determination coefficients (*r*^2^ > 0.94), as well as a good agreement between the adjusted and predicted determination coefficients shows good agreement of the experimental values and those expected by the selected models.

### 2.2. Optimization of Extraction Parameters and Model Validation

The values of independent variables best suited for the production of extracts with the highest TP contents are presented in Table 5. As expected from the quadratic equations presented in Table 3, moderate glycerol content and higher temperature beneficially affected TP concentration in glycerol extraction. On the other hand, low PPG content and relatively high temperature were the most suitable combination for PPG extraction. Even though extraction time did not exert statistically significant influence on the extraction, due to the observed slightly positive influence, higher extraction times were selected for both types of extraction. According to the calculations of optimal conditions for GL and PPG extraction, two extracts, GL-opt and PPG-opt were prepared and the TP content of the extracts determined. The predicted values, as well as the observed ones, are presented in Table 5. 

As presented in Table 5, the observed TP values in both extracts were in good accordance with the predicted ones. As expected, TP content in GL-opt was significantly higher than in PPG-opt. This confirms excellent ability of GL/water mixtures to dissolve plant phenolics, comparable to that of ethanol/water solutions [20].

### 2.3. Chemical Composition of the Extracts

Besides TP, total flavonoid (TF) and total phenolic acid content (TPA) in extracts prepared at optimal conditions was also assessed and presented. In addition, the presence of main phenolic acids and flavonoids aglycones, as well as phytoestrogens was analyzed by means of HPLC analysis (Table 6). 

The content of total flavonoids, as presented in Table 6, was significantly higher in GL-opt than in PPG-opt. Phenolic compounds in plant material are commonly present in form of glycosides or esters, respectively. Analysis of all the derivatives may be demanding and, in case of the lack of appropriate standard, their concentration may be underestimated. However, the type and quantity of the phenolic aglycones is the dominant features that determines pharmacological effect of the phenolics [39]. The results of HPLC analysis, preformed upon acid hydrolysis, revealed the presence of four flavonoid aglycones, apigenin, kaempferol, luteolin and quercetin. Their presence has previously been noted in samples of *M. lupulina* collected in Poland [26]. GL-opt contained notably higher content of luteolin and apigenin aglycones than PPG-opt. On the other hand, content of quercetin and kaempferol aglycones in GL-opt and PPG-opt, was rather similar. 

Spectrophotometric analysis has shown that the TPA content of two extracts prepared at the optimized conditions did not statistically differ (Table 6). HPLC analysis, on the other hand, revealed the slight differences in the content of two investigated phenolic acids, caffeic and ferulic acid. While this is the first report on the phenolic acids in *M. lupulina*, it is interesting to note that *M. sativa*, a related plants from Fabaceae family, also contained ferulic acid as one of the main phenolic constituents [40]. 

Butkute et al. [29] revealed the presence of several phytoestrogens in *M. lupulina*. As presented in Table 6, coumestrol, one of the most potent phytoestrogens and an agonist of α- and β-estrogen receptors [41], was found in our sample. In addition to estrogenic activity, coumestrol may offer photoprotective effects and down-regulate melanin production [9]. Even though the presence of biochanin A and formononetin was previously reported by Butkute et al. [29], they were not detected in this study. In addition, several other analyzed flavonoids (including flavones, flavonols and isoflavonoids) aglycones (chrysin, myricetin, daidzein, genistein and glycitein), as well as several phenolic acids (sinapic acid, *p*-coumaric acid, syringic acid, vanillic acid) were not detected in the analyzed extracts. 

**Figure A1:**
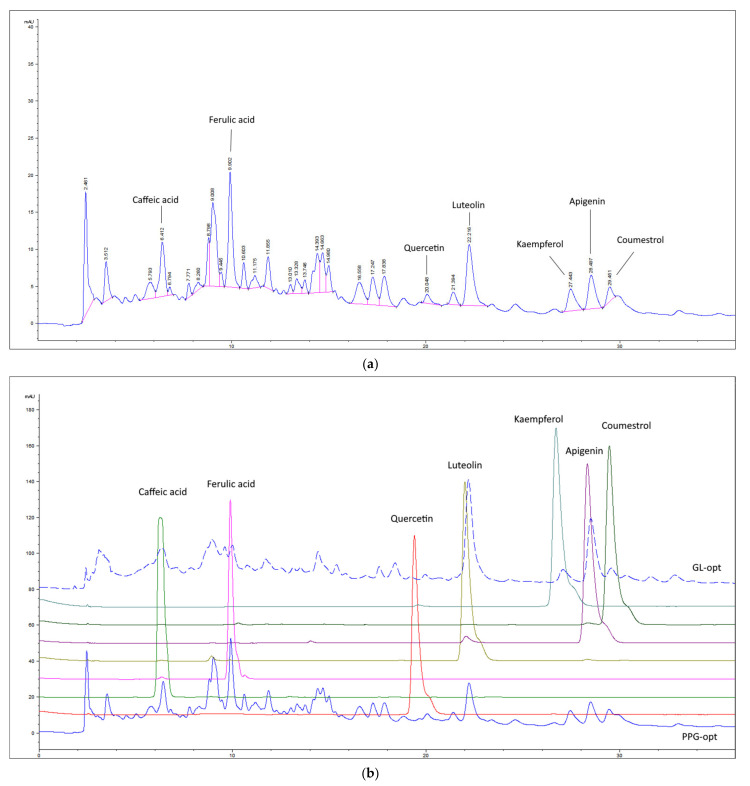
An example of **a**) chromatogram with peak annotations of the detected compounds (PPG-opt after 100 °C hydrolysis recorded at 320 nm using the method for determination of flavonoids and phenolic acids; details in Materials and Methods section) and **b**) chromatograms of PPG-opt, GL-opt and the phenolic standards in 2D signal overlay recorded in the same HPLC sequence.

A sample chromatogram where the peaks of all the detected compounds are visible is presented in Figure A1. It is evident that the detected phenolic compounds represent only a small part of phytochemical constituents of *M. lupulina*. While comprehensive metabolome analysis of *M. lupulina* was not a subject of this study, future, more in-depth research, using e.g., liquid chromatography coupled with mass spectrometry, will hopefully shed more light on the chemical composition of this plant species. 

### 2.4. Antioxidant Activity of the Extracts

**Figure 2:**
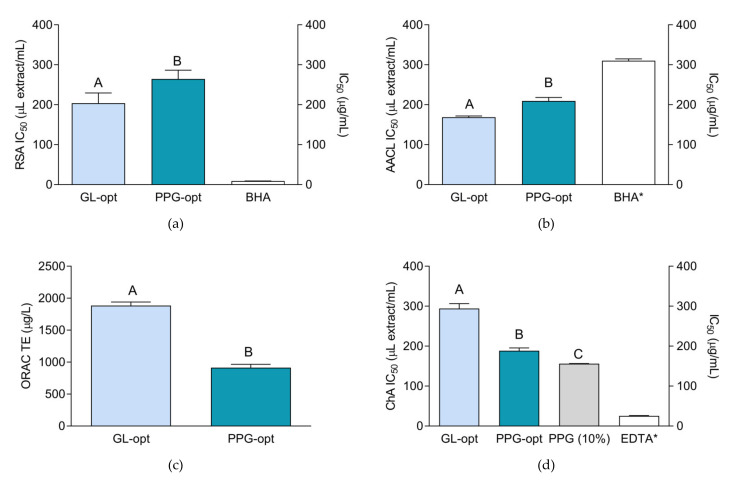
Antioxidant activity of the extracts and positive controls: BHA (butylated hydroxyanisole) and EDTA (ethylenediaminetetraacetic acid): (**a**) radical scavenging activity (RSA); (**b**) the antioxidant activity in β-carotene-linoleic acid assay (AACL); (**c**) activity in the ORAC assay expressed as trolox equivalents (TE) and (**d**) chelating activity (ChA). The activity is expressed as IC_50_ value (**a**,**b**,**d**) and Trolox equivalents (TE) (**c**). Asterisk (*) indicates that the IC_50_ unit is placed on the right *y*-axis. Different uppercase letters indicate statistically significant difference (*p* < 0.05).

Antioxidant activity of the extracts is presented in Figure 2. BHA, Trolox and EDTA were used as positive controls. They are commonly used as antioxidants (BHA, Trolox) or ion chelators (EDTA) in the pharmaceutical and cosmetic products. In addition, they are commonly used as positive references for antioxidant and chelating assays [42]. It is important to note that the activity of the extracts and the standards depicted in the Figure 2 may not be directly statistically compared because it is expressed in different measurements units (the activity of the extracts and standards was expressed as μL/mL and μg/mL, respectively). Nevertheless, the presented activity of the positive controls may be regarded as volume equivalents of 1 mg/mL solutions and is thus displayed for comparison purposes. 

The antioxidant activity of the prepared extracts, although relatively mild in comparison to the standards’ effectiveness (Figure 2), was visibly pronounced. With the exception of the chelating assay, GL-opt showed somewhat stronger activity in the performed antioxidant assays. With regard to phytochemical composition, and larger amount of almost all the investigated groups of phenolic compounds (TP, TF), as well as individual flavonoids, that have potent antioxidant activities [10], this finding is rather expected.

In order to establish if the extraction solvents influence the activity at the tested concentrations, their activity was tested in parallel with the extracts. While the solvents exerted no influence on the majority of the applied antioxidant assays, and are thus omitted from the figures, PPG showed a degree of activity in the chelating assay. Even though the detailed investigation of the mechanism of the observed effect is out of scope of this work, it would be interesting to determine whether the observed activity is due to the inherent ability of PPG to chelate Fe^2+^ ions or is it caused by some other effect e.g., the interaction of PPG with ferrozine.

### 2.5. Tyrosinase-, Elastase-, Lipoxygenase and Coagulation Inhibiting Activity

**Figure 3:**
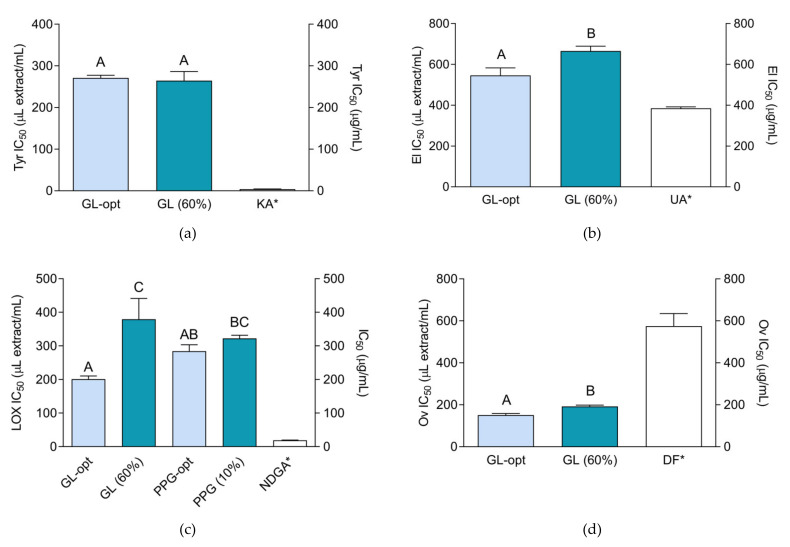
Cosmeceutical activity of the extracts, extraction solvents (60% glycerol-GL(60%)) and positive controls: kojic acid (KA), ursolic acid (UA), nordihydroguaiaretic acid (NDGA) and diclofenac (DF): (**a**) tyrosinase-inhibiting activity (Tyr); (**b**) elastase-inhibiting activity (El); (**c**) lipoxigenase-inhibiting activity (LOX); (**d**) ovalbumin coagulation-inhibiting activity (Ov). The activity is expressed as IC_50_ value. Asterisk (*) indicates that the IC_50_ unit is placed on the right *y*-axis. Different uppercase letters indicate statistically significant difference (*p* < 0.05).

The results, presented in Figure 3, show that GL-opt displayed various degrees of the activity in all the performed assays. While both GL and GL-opt were active in all assays, the activity of both PPG-opt and PPG in most assays was negligible.

As presented in Figure 3, the IC_50_ value of GL-opt in tyrosinase inhibiting assay was lower than the corresponding activity of 1 mg/mL kojic acid solution. Furthermore, the extraction solvent, 60% glycerol, displayed statistically equal levels of the activity and thus was the most responsible for the observed activity. This finding is somewhat unexpected due to the presence of coumestrol, coumestan with anti-tyrosinase properties, in GL-opt. It is possible that relatively low amounts found in the prepared extracts were not sufficient to display the desired activity.

Elastase-inhibiting activity of GL-opt was higher than the activity of the extraction solvent indicating a synergistic or additive influence of phytochemical components of the extract and the extraction solvent. Furthermore, the activity of GL-opt was lower but comparable to ursolic acid indicating a relatively good activity. A study investigating the refirming effect of a plant complex containing plants from Fabaceae family, including *M. sativa*, has shown a significant anti-elastase potential of the plant mixture, and the effects were confirmed *in vivo*. However, as the investigated preparation was a mixture it is difficult to assess how much the activity was influenced by the individual components [43]. 

Among the investigated samples, GL-opt demonstrated the strongest anti-LOX activity, surpassing the activity of the solvent alone. Nevertheless, the activity was still lower than the activity of 1 mg/mL NDGA solution. PPG-opt demonstrated anti-LOX properties, although the observed activity was mostly due to the extraction solvent, whose activity did not statistically differ. Unlike the previous assays, coagulation-inhibiting activity of GL-opt was rather strong and favorably comparable to the activity of 1 mg/mL diclofenac solution. Similar to the activity in elastase-inhibiting assay, the glycerol/water mixtures were also interfering with the enzyme activity. However, the activity of the extract surpassed the activity of the extraction solvent confirming that this activity is enhanced by the extracted secondary metabolites. This indicates that *M. lupulina* exerts anti-inflammatory activity and that its glycerolic preparations may be a potentially valuable ingredients of food supplements for the prevention of inflammatory disorders. Even though it is sometimes not possible to compare the results of the different anti-inflammatory activity assays due to different models and conditions, it is interesting to note that *M. sativa*, medically the most important plant from *Medicago* genus, also shows a significant anti-inflammatory potential. For example, hydroalcoholic extracts of *M. sativa*, have demonstrated analgesic and anti-inflammatory properties in a rat model of inflammation [44]. Furthermore, ethyl acetate extracts of the same plant inhibited lipopolysaccharide-induced inflammation in vitro and in vivo [45]. 

Low activity of PPG-opt is rather surprising because the cosmeceutical activities of flavonoids and phenolic acids that it contains have been demonstrated in previous studies. For example, phytochemical and bioactivity studies of the aerial parts of *Persicaria senticosa* have shown that its constituent quercetin is a potent tyrosinase inhibitor [46]. Furthermore, methylated derivatives of quercetin and kaempferol were able to inhibit neutrophil elastase [47], while ferulic acid is considered to be one of the leading cosmeceuticals for facial hyperpigmentation [48]. It is possible that better activity of glycerol extract, besides the significant solvent contribution, is partly related to the higher amount of phenolic substances in the extracts and/or other substances whose quantity was not determined in course of this study. 

### 2.6. α-Glucosidase- and α-Amylase-Inhibiting Activity

**Figure 4:**
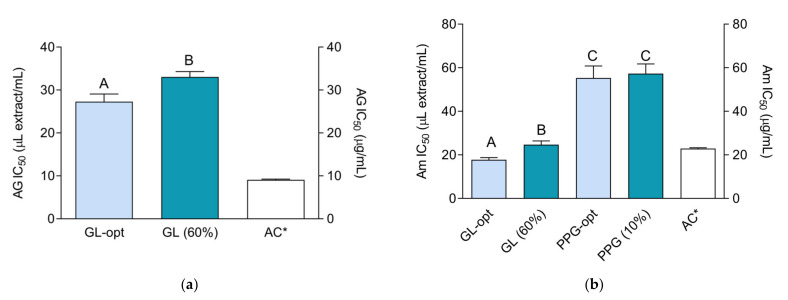
Antidiabetic activity of the extracts and positive control acarbose (AC): (**a**) α-glucosidase-inhibiting activity (AG); (**b**) α-amylase-inhibiting activity (Am). The activity is expressed as IC_50_ value. Asterisk (*) indicates that the IC_50_ unit is placed on the right *y*-axis. Different uppercase letters indicate statistically significant difference (*p* < 0.05).

As presented in Figure 4, GL-opt extracts exerted influence over both enzymes, while PPG-opt influenced only α-amylase. Similar to the activity in the previous assays, a portion of the activity that GL-opt exerted on α-glucosidase was due to the extraction solvent. However, the phytochemical components of the extract also influenced the observed activity as demonstrated in significant differences between the activity of the extraction solvent and the extract. Coumestrol and luteolin were previously found to be potent α-glucosidase inhibitors [49,50]. Thus, it is possible that the observed difference in activity may, at least partly, be related to their higher content in GL-opt. Both GL-opt and PPG-opt displayed a notable activity in the anti-amylase assay. However, while the activity of GL-opt was similar to the activity of 1 mg/mL acarbose, the activity of PPG-opt was significantly lower. In addition, the anti-amylase activity of PPG-opt was entirely due to extraction solvent. 

Interestingly, even though *M. sativa*, a related and well know phytotherapeutic plant, is traditionally used as an anti-diabetic agent [51], its α-glucosidase- and α-amylase-inhibitory properties were not investigated. Similar to the previous assays, the better activity of GL-opt in comparison with PPG-opt in these assays may be explained by the higher phenolic and flavonoid content of the extract. For example, various phenolic acids and flavonoids, including quercetine and caffeic acid, may strongly suppress the activity of the enzymes included in carbohydrate digestion [52]. In addition, current studies suggest that combinations of plant phenolics may have an additive effect on *α*-glucosidase inhibition [53] granting more potent activity of the plant extract in comparison with pure phytochemicals. 

## 3. Materials and Methods

### 3.1. Plant Materials and Chemicals 

Flowering above-ground parts of *M. lupulina* were collected near Lake Jarun, in the surroundings of Zagreb (45°48′ N 15°90′ E). The producer of PPG 425 was A&C (Clonmel, Ireland). Purity of standards (acarbose, BHA, diclofenac, EDTA, kojic acid, nordihydroguaiaretic acid (NDGA) and 6-hydroxy-2,5,7,8-tetramethylchroman-2-carboxylic acid (Trolox)) was ≥ 98.5%. The standards and the enzymes (α-glucosidase from *Saccharomyces cerevisiae*, α-amylase and elastase from porcine pancreas, mushroom tyrosinase and soybean LOX) were purchased from Sigma-Aldrich (St. Louis, MO, USA). Other reagents and chemicals were of analytical grade. The plant material was identified by Vedran Šegota, expert associate of Herbarium Croaticum at Division of Botany, Department of Biology, Faculty of Science, University of Zagreb. Voucher specimen is deposited in the Department of Pharmacognosy, University of Zagreb Faculty of Pharmacy and Biochemistry, Zagreb, Croatia (FG-2019-ML).

### 3.2. Preparation of Extracts

Aerial parts of *M. lupulina* consisting of flowers, leaves and stems were dried in a well-ventilated room without direct sunlight for 14 days at 23 ± 2 °C. Aliquots (0.1 g) of plant material, previously grinded and passed through a sieve of 850 μm mesh size, were suspended in 10 mL of the appropriate solvent in a 50 mL Erlenmeyer flask. The flasks were placed in an ultrasonic bath (SONOREX^®^ Digital 10 P DK 156 BP, Bandelin, Berlin, Germany) at 360 W set to the appropriate temperature. After the extraction and the subsequent filtration (using Filtrak Qualitative Folded Filters Grade 6, 80 g/m^2^, Sartorius, Göttingen, Germany) the extracts were stored at −20 °C in the dark until use. The detailed extraction conditions, that specify the solvent composition, temperature and extraction time for each extract, are presented in Table 2. 

### 3.3. Experimental Design 

The preparation of the two BBDs, regression analysis and optimization of the results was performed using Design Expert software version 8.0.6 (Stat-Ease, Minneapolis, MN, USA). The range of values for the three independent variables, as well as the corresponding codes, were chosen as presented in Table 1. TP was selected as the response (Table 2). Experimental data were fitted to a quadratic polynomial model as described in:

(1) $${Y=A}_{0}+\sum_{i=1}^{k}A_{i}X_{i}\;+\sum_{i=1}^{k}A_{\mathrm{ii}}X_{i}^{2}+\sum_{i=1}^{k-1}\times \sum_{j=1+1}^{k}A_{\mathrm{ij}}X_{i}X_{j},$$

where Y is the dependent variable; A_0_, A_i_, A_ii_, and A_ij_ are the regression coefficients for intercept, linearity, square and interaction, respectively; Xi and Xj are the independent variables. 

### 3.4. Total Phenol Content 

Total phenol content (TP) was determined using Folin–Ciocalteau reagent [54]. To the mixture of the extract (40 μL), water (40 μL) and Folin Ciocalteu reagent (80 μL), 80 μL of Na_2_CO_3_ solution (1 %) was added. After 1 h at room temperature, the absorbance was measured at 660 nm. TP content was calculated from the calibration curve of gallic acid and expressed as μg of gallic acid equivalents (GAE) in mL of the extract.

### 3.5. Total Flavonoid Content 

For determination of total flavonoid content (TF) the reaction with aluminum chloride was used [55]. Extract (120 μL) and AlCl_3_ solution (120 μL, 0.2% in methanol) were mixed. After 1 h at the absorbance was measured at 420 nm after. TF content was calculated from the calibration curve of quercetin and expressed as μg of quercetin equivalents (QE) in mL of the extract.

### 3.6. Total Phenolic Acid Content 

TPA content was assessed with nitrite-molybdate reagent [56]. To 100 µL of the extract, 50 µL of 0.5 M HCl and 50 µL of the nitrite-molybdate reagent (prepared by dissolving 10 g of sodium nitrite and 10 g of sodium molybdate in 100 mL of water) were added, followed by addition of 50 µL of 8.5% NaOH. The absorbance at 492 nm was measured. TPA content was calculated from the calibration curve of caffeic acid and expressed as μg of caffeic acid equivalents (CAE) in mL of the extract.

### 3.7. HPLC Analysis of Phenolic Constituents

For determination of individual phenolics, an Agilent 1200 series HPLC instrument (Agilent Technologies, Santa Clara, CA, USA) equipped with an autosampler, DAD detector, and a Zorbax Eclipse XDB-C18 (5 μm, 12.5 mm × 4.6 mm) column was used. For flavonoid aglycones and free acids determination, in 1 mL of the corresponding extract solution 400 μL 6M HCl was added. The obtained mixtures were heated for 2 h at boiling temperature (for determination of flavones) or 80 °C (for determination of flavonols and phenolic acids) in water bath and then filtered to 5 mL volumetric flask. The precipitate on filter paper was washed with methanol and added to the flask contents to the volume. The extracts and the phenolic standards (0.2 mg/mL) were filtered through a syringe filter (PTFE with 0.45 μm pore size). For determination of flavonoids and phenolic acids, HPLC chromatographic separation was performed at 40 °C and 1.0 mL/min flow rate. The mobile phases A and B, consisting of water, methanol, and formic acid in proportions 93:5:2 (*v*:*v*:*v*) and 3:95:2 (*v*:*v*:*v*), respectively, were applied in the following order: 0 min 20% B, 10 min 40% B, 35 min 50% B. For coumestrol determination, gradient elution was carried out with water:acetic acid (99.9:0.1) and acetonitrile as solvent A and B, respectively. The gradient elution program was as follows: 0–5 min, 5–40% B; 5–15 min, 40–15% B; 15–25 min, 15–30% B; 25–35 min, 30–40% B; 35–45 min, 40–50% B; 45–55 min, 50–95% B; 55–65 min, 95–5% B with flow rate 1.0 mL/min [49]. For calibration curve preparation, varying volumes of standard solutions were injected using an autosampler. Apigenin, kaempferol, luteolin and quercetin were quantified at 270 nm, while coumestrol was quantified at 254 nm. For caffeic and ferulic acid, absorbance at 320 nm was recorded. The peak assignment and identification were based on comparison of retention times of peaks in sample chromatogram and UV spectra (200–500 nm) with those of the standards. Calibration curve parameters, level of detection (LOD), and level of quantification and (LOQ) are reported in Table 7. The calibration curves for the standards employed in this work that were not detected in the analysed samples (sinapic acid, *p*-coumaric acid, syringic acid, vanillic acid, chrysin, myricetin, daidzein, genistein, glycitein, formononetin and biochanin A) are not reported.

### 3.8. Free Radical Scavenging Activity 

For determination of radical scavenging activity (RSA) methanolic DPPH solution (70 μL, 0.21 mg/mL) and 130 μL of either the methanolic solution of the extract (sample), methanol (negative control) or BHA (positive control) were mixed and left in the dark at room temperature for 30 min [40]. The absorbance was read at 545 nm using microplate reader (FLUOstar Omega, BMG Labtech, Ortenberg, Germany). RSA was calculated as follows:

(2) $$\mathrm{RSA}\;\left(\%\right)=\frac{A_{control}-A_{sample}}{A_{control}}\times 100$$

where A_control_ is the absorbance of the negative control and A_sample_ is the absorbance of the DPPH solution with the corresponding extract. RSA IC_50_, concentration of the extract that scavenges 50% of DPPH free radicals present in the solution, was expressed as μL of extract/mL of solution (μL extract/mL). 

### 3.9. Fe^2+^ Chelating Activity

For determination of chelating activity (ChA), the extracts (130 μL) and FeSO_4_ (30 μL, 0.22 mM) were mixed and left to stand in the dark. After 5 min, ferrozine solution (50 μL, 1.0 mM) was added [57]. Following additional 30 min, the absorbance was recorded at 562 nm. Reaction mixture containing water or EDTA solution (130 μL) instead of the extract served as the negative and positive control, respectively. ChA was calculated as follows

(3) $$ChA\;\left(\%\right)=\frac{A_{control}-A_{sample}}{A_{control}}\times 100$$

where A_control_ is the absorbance of the negative control and A_sample_ is the absorbance of the respective extract. ChA IC_50_ was calculated as the concentration of the extract which chelates 50% of Fe^2+^ present in the solution and expressed as μL of extract/mL of solution (μL extract/mL). 

### 3.10. Antioxidant Activity in β-Carotene-Linoleic Acid Assay

Reaction mixture for determination of antioxidant activity in β-carotene-linoleic acid assay (AACL) [58] consisted of 200 μL of the aqueous emulsion with β-carotene (6.7 μg/mL), linoleic acid (0.7 mg/mL) and Tween 40 (6.7 mg/mL) and 50 μL of the extract solution in methanol heated at the temperature of 50 °C. Reaction mixture containing methanol or BHA solution (50 μL) instead of the extract served as the negative and positive control, respectively. The antioxidant activity in β-carotene linoleic acid assay (AACL) was calculated based on the absorbance recorded at 470 nm after 60 min was calculated as follows:

(4) $$AACL\;\left(\%\right)=\frac{A_{sample}}{A_{control}}\times 100$$

where A_control_ and A_sample_ are the absorbances of the water control and the antioxidant, respectively. AACL IC_50_ was calculated as the concentration of the extract that protects 50% β-carotene present in the solution and expressed as μL of extract/mL of solution (μL extract/mL). 

### 3.11. ORAC

For the oxygen radical absorbance capacity (ORAC) assay [59], 25 μL of the extract solution or phosphate buffer (blank), and 150 μL of fluorescein (1 μM) were preincubated 37 °C. After 10 min, 25 μL of 2,2’-azobis(2-amidinopropane) dihydrochloride (AAPH) (125 mM) was added and the fluorescence were measured kinetically at 37 °C every 150 s for 60 min. The excitation and emission wavelengths were 485 nm and 528 nm, respectively. The ORAC activity of a sample was calculated by subtracting the area under the blank curve from the area under the sample curve to obtain the net area under the curve. Using Trolox of known concentration, a standard curve was generated and the ORAC activity of the samples was calculated as Trolox equivalents.

### 3.12. Tyrosinase Inhibitory Activity 

For tyrosinase inhibition determination (TyInh) [60], 120 μL extract solution and 40 μL of tyrosinase solution dissolved in 16 mM pH 6,8 phosphate buffer were mixed and kept in dark at room temperature. After 10 min, L-DOPA solution (60 μL, 0.19 mg/mL) in the same buffer was added. The absorbance was measured at 492 nm after 10 min. Reaction mixture containing buffer or kojic acid solution (80 μL) instead of the extract served as the negative and positive control, respectively. TyInh was calculated as follows:

(5) $$TyInh\;\left(\%\right)=\frac{A_{control}-A_{sample}}{A_{control}}\times 100$$

where A_control_ is the absorbance of the negative control and A_sample_ is the absorbance of the respective extract. TyInh IC_50_ was calculated as the concentration of the extract which inhibits 50% of tyrosinase activity and expressed as μL of extract/mL of solution (μL extract/mL). 

### 3.13. Elastase Inhibitory Activity 

For elastase inhibitory activity determination [61], 100 μL of plant extract solution in Tris-HCl buffer (0.1 M, pH 8.0) was added to 1 mM *N*-succinyl-(Ala)_3_-nitroanilide (SANA) in the same buffer. After 10 min at 25 °C, 25 µl of elastase solution was added. The absorbance was measured at 410 nm after additional 10 min. Reaction mixture containing buffer or ursolic acid solution (100 μL) instead of the extract served as the negative and positive control, respectively. Elastase inhibitory activity (ElInh) was calculated as:

(6) $$ElInh\;\left(\%\right)=\frac{A_{control}-A_{sample}}{A_{control}}\times 100$$

where A_control_ is the absorbance of the negative control and A_sample_ is the absorbance of the respective extract. Concentration of the extract, which inhibits 50% of elastase activity (ElInh IC_50_), was calculated and expressed as μL of extract/mL of solution (μL extract/mL). 

### 3.14. Lipoxygenase Inhibitory Activity 

For LOX inhibitory activity [62], 100 µL of extract solution, 30 μL of LOX solution and 30 μL of linoleic acid in phosphate buffer (pH 8, 100 μM) were mixed and incubated at 25 °C. After 10 min the absorbance was determined at 234 nm. Reaction mixture containing buffer or NDGA acid solution (100 μL) instead of the extract served as the negative and positive control, respectively. LOX inhibitory activity (LOXInh) was calculated as follows:

(7) $$LOXInh\;\left(\%\right)=\frac{A_{control}-A_{sample}}{A_{control}}\times 100$$

where A_control_ is the absorbance of the negative control and A_sample_ is the absorbance of the respective extract. LOXInh IC_50_ was calculated as the concentration of the extract which inhibited 50% of LOX activity and expressed as μL of extract/mL of solution (μL extract/mL). 

### 3.15. Inhibition of Heat-Induced Protein Coagulation 

For determination of anti-inflammatory activity in the heat-induced ovalbumin coagulation method [16], 30 μL of ovalbumin solution and 160 μL of the extract solution in phosphate buffered saline (pH 6.4) were mixed. After 15 min at 37 °C, the mixture was heated at 80 °C. After 5 min the absorbance at 660 nm was recorded. Reaction mixture containing buffer or diclofenac sodium solution (50 μL) instead of the extract served as the negative and positive control, respectively. The percentage inhibition of ovalbumin denaturation (OvInh) was calculated as follows:

(8) $$OvInh\;\left(\%\right)=\frac{A_{control}-A_{sample}}{A_{control}}\times 100$$

where A_control_ is the absorbance of the negative control and A_sample_ is the absorbance of the respective extract. OvInh IC_50_ was calculated as the concentration of the extract which inhibits 50% of the ovalbumin coagulation and expressed as μL of extract/mL of solution (μL extract/mL). 

### 3.16. α-Glucosidase Inhibition Assay

For *α*-glucosidase determination [57] 20 μL of extract solution and 50 μL of *α*-glucosidase solution in 0.1 M phosphate buffer (pH 6.8) were incubated for at 37 °C. After 10 min 50 μL of 1 mM *p*-nitrophenyl-*α*-d-glucopyranoside, dissolved in the same buffer, was added and the absorbance recorded at 405 nm. Reaction mixture containing buffer or acarbose solution (20 μL) instead of the extract served as the negative and positive control, respectively. Percentage of *α*-glucosidase inhibition (*AglInh*) was calculated as follows:

(9) $$AglInh\;\left(\%\right)=\frac{A_{control}-A_{sample}}{A_{control}}\times 100$$

where *A_control_* is the absorbance of the negative control and *A_sample_* is the absorbance of the reaction mixture containing extracts. Concentration of the extract which inhibits 50% *α*-glucosidase activity (*AglInh* IC_50_) was calculated and expressed as μL of extract/mL of solution (μL extract/mL). 

### 3.17. α-amylase Inhibition Assay

For α-amylase inhibition assay [63]. 0.5 mL of the extract and 0.5 mL of α-amylase (0.8 U/mL) in 20 mM phosphate buffer (pH 6.9) were preincubated at 25 °C. After 10 min 0.5 mL of soluble starch (0.5% solution in the same buffer) was added. After additional for 10 min the reaction was stopped with 1 mL of 96 mM 3.5-dinitrosalicylic acid color reagent and the test tubes placed in a boiling water bath for 5 min. Upon cooling to room temperature, the reaction mixtures were diluted by adding 10 mL distilled water and absorbance was measured at 540 nm. Reaction mixture containing buffer or acarbose solution (20 μL) instead of the extract served as the negative and positive control, respectively. The percentage of α-amylase inhibition (AmInh) was calculated as follows

(10) $$AmInh\;\left(\%\right)=\frac{A_{control}-A_{sample}}{A_{control}}\times 100$$

where A_control_ is the absorbance of the negative control and A_sample_ is the absorbance of the reaction mixture containing extracts. Concentration of the extract which inhibits 50% amylase activity (AmInh IC_50_) was calculated and expressed as μL of extract/mL of solution (μL extract/mL). 

### 3.18. Statistical Analysis

The measurements are performed in triplicate and the IC_50_ values were calculated using regression analysis. The results are presented as mean ± standard deviation. Statistical comparisons were made using one-way ANOVA, followed by Tukey’s post-hoc test for multiple comparisons (GraphPad Prism, San Diego, CA, USA). *p*-values < 0.05 were considered statistically significant. 

## 4. Conclusions

Two non-toxic organic solvents with humectant properties, PPG and GL, were found to be suitable solvents for UAE extraction of phenolic constituents from *M. lupulina*. The extracts prepared according to the procedure optimal for extraction of phenolics (GL-opt and PPG opt) were efficient antioxidants, radical scavengers and Fe^2+^ ion chelators. Furthermore, they were able to impair heat-induced degradation of linoleic acid, as well as and LOX and α-amylase activity. In addition to that, GL-opt was efficient inhibitor of protein coagulation, as well as anti-elastase and anti-α-glucosidase agent. Albeit GL-opt showed a moderate anti-tyrosinase activity, it was established that the observed activity was due to the action of the extraction solvent. The PPG/water mixtures were suitable for extraction of phenolic compounds from *M. lupulina* and preparation of extract with antioxidant properties. GL-opt, on the other hand, yielded additional benefit of having the extraction solvent that was active in most of the performed assays. Thus, the optimized glycerol extracts is may be regarded as an suitable candidates for production of food supplements and topical products with antidiabetic and cosmeceutical properties. 

## Figures and Tables

**Table 1:** Levels of extraction variables for the Box–Behnken designs.

Independent Variables	Code	Levels		
		−1	0	1
Glycerol/PPG Concentration (%, *w*/*w*)	X_1_	10	50	90
Temperature (°C)	X_2_	20	50	80
Time (min)	X_3_	20	40	60

**Table 2:** The Box Behnken design and content of total phenols (TP) in the extracts in glycerol (GL) and polypropylene glycol (PPG) extraction.

Standard Order	Run in GL	Run in PPG	X_1_ (%. *w*/*w*)	X_2_ (°C)	X_3_ (min)	TP in GL	TP in PPG
						μg/mL	μL/mL
1	8	5	10	20	40	184.52	140.09
2	4	13	90	20	40	98.86	48.07
3	16	14	10	80	40	190.61	167.71
4	11	1	90	80	40	197.03	68.09
5	12	10	10	50	20	184.52	146.72
6	17	16	90	50	20	177.69	62.59
7	5	6	10	50	60	230.16	143.21
8	3	8	90	50	60	188.23	60.61
9	7	12	50	20	20	194.49	113.99
10	10	4	50	80	20	244.87	128.40
11	1	2	50	20	60	208.56	119.95
12	6	3	50	80	60	247.77	140.20
13	14	17	50	50	40	216.15	127.72
14	2	9	50	50	40	248.12	106.67
15	15	7	50	50	40	234.51	127.66
16	13	15	50	50	40	220.61	124.60
17	9	11	50	50	40	228.19	96.12

X_1_ = GL/PPG content, X_2_ = temperature, X_3_ = time.

**Table 3:** Polynomial equations for the total phenolic content (TP) in glycerol (GL) and polypropyleneglycol (PPG) extraction models.

Response	Unit	The Equation Coefficients: a×X_1_^2^ + b × X_2_^2^ + c × X_3_^2^ + d × X_1_ × X_2_ + e × X_1_ × X_3_ + f × X_2_ × X_3_ + g × X_1_ + h × X_2_ + i × X_3_ + j									
		a	b	c	d	e	f	g	h	i	j
TP in GL	(μg/mL)	−45.3 *	−16.5 *	10.9	23.0*	−8.8	−2.8	−16.0 *	24.2*	9.1	229.5
TP in PPG	(mL/mL)	−16.5 *	5.9	3.2	−1.9	0.4	1.5	−44.8 *	10.3 *	1.5	116.6

* = The significant equation terms (*p* < 0.05). X_1_ = GL/PPG content, X_2_ = temperature, X_3_ = time.

**Table 4:** Analysis of variance (ANOVA) for the fitted model quadratic polynomial equations.

	TP-GL *r*^2^ = 0.9545; *r*^2^_adj_ = 0.8959; *r*^2^_pr_ = 0.7008					TP-PPG *r*^2^ = 0.9483; *r*^2^_adj_ = 0.8817; *r*^2^_pr_ = 0.7942				
Source	SS	df	MS	F Value	*p*-Value	SS	df	MS	*F* Value	*p*-Value
Model	20240	9	2248	16.3	0.0007	18218	9	2024	14.25	0.0010
Lack of Fit	335.0	3	111.7	0.708	0.5955	166.3	3	55.41	0.2677	0.8462
Pure Error	630.8	4	157.7			828.0	4	207.0		

*r*^2^_adj_ = adjusted *r*^2^; *r*^2^_pr_ = predicted *r*^2^; SS = sum of squares; df = degrees of freedom; MS = mean square.

**Table 5:** Predicted and observed responses values for the extracts prepared at optimal conditions.

Extract Name	Optimized Response	Aim of the Optimization	X_1_ °C	X_2_ %	X_3_ W	Predicted	Observed	RD (%)
GL-opt	TP (μg/mL)	maximized	45	70	60	255.9	256.0± 10.5^A^	+1.62
PPG-opt	TP (μg/mL)	minimized	10	80	60	168.4	162.5± 1.8^B^	−3.50

X_1_ = GL/PPG content, X_2_ = temperature, X_3_ = time; RD = Response deviation, calculated as (Observed-Predicted)/Predicted ×100; ^A,B^ = Different uppercase letters indicate statistically significant difference (*p* < 0.05).

**Table 6:** Content of total flavonoids (TF), total phenolic acids (TPA), individual phenolic acids and flavonoid aglycones in *M. lupulina* extracts.

Extract	TF (μg/mL)	TPA (μg/mL)	Api (μg/mL)	Kae (μg/mL)	Lut (μg/mL)	Que (μg/mL)	CA (μg/mL)	FA (μg/mL)	Cou (μg/mL)
GL-opt	94.62 ± 5.5^A^	41.01 ± 3.03^A^	5.07	5.58	13.39	5.50	3.88	4.22	3.42
PPG-opt	77.96 ± 4.28^B^	40.25 ± 1.77^A^	1.44	5.34	4.80	5.45	2.42	6.98	1.52

Api = Apigenin, Kae = Kaempferol, Lut = Luteolin, Que = Quercetin, CA = Caffeic acid, FA = Ferulic acid, Cou = Coumestrol; ^A,B^ = Different uppercase letters indicate statistically significant difference (*p* < 0.05).

**Table 7:** Calibration curve parameters for selected phenolic standards.

Standard	Equation	*r* ^2^	LOD (μg/mL)	LOQ (μg/mL)
Apigenin	y = 3755.16 x − 27.75	0.9998	0.028	0.085
Kaempferol	y = 2802.76 x − 21.62	0.9998	0.025	0.078
Luteolin	y = 2787.17 x + 46.60	0.9998	0.025	0.076
Quercetin	y = 2200.20 x − 36.75	0.9998	0.027	0.083
Caffeic acid	y = 5335.00 x − 19.45	0.9999	0.012	0.041
Ferulic acid	y = 5045.04 x − 45.10	0.9998	0.026	0.088
Coumestrol	y = 2759.54 x − 44.60	0.9997	0.076	0.2298

LOD = level of detection; LOQ = Level of quantification; y = Area under curve (mAU×s); x = amount of the standard (μg).

## Data Availability

The data presented in this study are available on request from the corresponding author.

## Sample Availability

Samples of the compounds are not available from the authors.

## Appendix A

In the chromatograms of PPG-opt after hydrolysis at 100 °C the peaks belonging to all the recorded standards may be discerned (Figure A1). It is important to note that the chromatograms are presented for illustration purposes only. The chromatograms used for the actual quantifications were done using several different sample preparation methods (two methods using different hydrolysis conditions, and one method without hydrolysis), different separation methods (one for flavonoids and phenolic acids and the other for coumestrol), as well as using different wavelengths, according to the standard (320 nm for phenolc acids, 270 for flavonoids, and 254 nm for coumestrol). Identity was confirmed based on both, the retention time and the UV-spectrum recorded in the region 200-500 nm.
